# Caffeic acid O-methyltransferase from *Ligusticum chuanxiong* alleviates drought stress, and improves lignin and melatonin biosynthesis

**DOI:** 10.3389/fpls.2024.1458296

**Published:** 2024-09-18

**Authors:** Enxia Huang, Jie Tang, Simin Song, Han Yan, Xinyi Yu, Chenlu Luo, Yineng Chen, Huiyue Ji, Anqi Chen, Jiayu Zhou, Hai Liao

**Affiliations:** School of Life Science and Engineering, Southwest Jiaotong University, Chengdu, Sichuan, China

**Keywords:** caffeic acid O-methlytransferase, lignin, melatonin, drought tolerance, *Apiaceae* family

## Abstract

Drought stress is a major constraint on plant growth and agricultural productivity. Caffeic acid O-methyltransferase (COMT), an enzyme involved in the methylation of various substrates, plays a pivotal role in plant responses to abiotic stress. The involvement of COMTs in drought response, particularly through the enhancement of lignin and melatonin biosynthesis, remains poorly understood. In this study, LcCOMT was firstly proposed to be associated with the biosynthesis of both lignin and melatonin, as demonstrated through sequence comparison, phylogenetic analysis, and conserved motif identification. *In vitro* enzymatic assays revealed that LcCOMT effectively methylates N-acetylserotonin to melatonin, albeit with a higher *K*m value compared to caffeic acid. Site-directed mutagenesis of residues Phe171 and Asp269 resulted in a significant reduction in catalytic activity for caffeic acid, with minimal impact on N-acetylserotonin, underscoring the specificity of these residues in substrate binding and catalysis. Under drought conditions, *LcCOMT* expression was significantly upregulated. Overexpression of *LcCOMT* gene in *Arabidopsis* plants conferred enhanced drought tolerance, characterized by elevated lignin and melatonin levels, increased chlorophyll and carotenoid content, heightened activities of antioxidant enzymes peroxidase (POD), catalase (CAT), and superoxide dismutase (SOD), and reduced malondialdehyde (MDA) and hydrogen peroxide (H_2_O_2_) accumulation. This study is among the few to demonstrate that COMT-mediated drought tolerance is achieved through the simultaneous promotion of lignin and melatonin biosynthesis. LcCOMT represents the first functionally characterized COMT in *Apiaceae* family, and it holds potential as a target for genetic enhancement of drought tolerance in future crop improvement strategies.

## 1 Introduction

The global demand for increased crop yield has significantly escalated to accommodate the continuous growth of the population in recent decades. Abiotic stresses represent significant adverse environmental conditions that impact plant growth, development, and productivity (Gong et al., 2020). Drought stands out as one of the most severe abiotic stresses that plants frequently face (Billah et al., 2021). The ongoing emission of greenhouse gases is leading to a warmer and drier climate, thereby increasing the projected intensity and frequency of drought events (Gadani and Vyas, 2011; Patz et al., 2014; Huang et al., 2023). Therefore, the comprehension and enhancement of plant responses to drought stress are crucial. Previous studies have revealed that drought stress triggers the excessive accumulation of reactive oxygen species (ROS) leading to the disruption of intracellular homeostasis and damage to cell membranes and other organelles (Fang and Xiong, 2015). Throughout plant evolution, various secondary metabolites have been synthesized to help plants combat drought stress (Lam et al., 2024). Among these, lignin, a phenolic polymer, plays a crucial role in providing mechanical support, facilitating tissue/organ development, and enabling efficient water and nutrient transport in plants (Liu et al., 2018; Vanholme et al., 2019; Qin et al., 2022). The accumulation of lignin significantly contributes to resistance against drought, salt, and bacterial wilt disease (Ma et al., 2017; Chun et al., 2019; Qin et al., 2022). Additionally, melatonin, a tryptophan-derived molecule presented in various organisms, aids in adapting to different stresses (Zhan et al., 2019; Li et al., 2022a). Melatonin acts as a potent antioxidant, regulates stress-responsive gene transcription, and modulates the biosynthesis of secondary metabolites for stress tolerance (Zhao et al., 2022a).

In higher plants, S-adenosyl-l-methionine (SAM)-dependent O-methyltransferases (OMTs) play a crucial role in the biosynthesis of various natural products, such as lignin, flavonoids, alkaloids, and anthraquinones (Li et al., 2015; Deng et al., 2018; Lu et al., 2022; Liao et al., 2023). Caffeic acid O-methyltransferase (COMT), classified as a group-2 OMT, is responsible for catalyzing the methylation of caffeic acid to produce ferulic acid, a key precursor in lignin synthesis (Li et al., 2015; Morris and Facchini, 2019). Additionally, COMTs can act as alternative enzymes for N-acetylserotonin methyltransferase (ASMT), leading to the production of melatonin and its precursors (Chang et al., 2021; Pham et al., 2024). Current research indicates that COMTs from the majority of plant species primarily catalyze melatonin production from N-acetylserotonin rather than 5-methoxytryptamine from serotonin, suggesting a specific pathway preference (Zhang et al., 2023). Notably, COMTs from *Brassica napus*, *Ligusticum chuanxiong*, and watermelon exhibit elevated mRNA and activity levels under conditions of drought and salinity stress, suggesting their involvement in stress response mechanisms (Li et al., 2015, 2016; Chang et al., 2021). The expression and activities of COMTs demonstrate a significant positive correlation with lignin levels and enhanced stress tolerance (Singh and Sharma, 2023; Lam et al., 2024). Conversely, a study on 13 *COMTs* from *Oryza sativa* revealed reduced mRNA levels under drought stress, indicating potential divergent functions among different plant species (Liang et al., 2022). Chang et al. (2021); Li et al. (2022b), and Yao et al. (2022) demonstrated that transgenic plants overexpressing *COMT* genes from watermelon, *Carex rigescens*, and *Nicotiana tabacum*, respectively, exhibit enhanced drought tolerance through increased melatonin levels. Application of exogenous melatonin has also been found to enhance the ability of plants to withstand abiotic stresses (Zhang et al., 2015). These results underscore the involvement of plant *COMT* genes in responding to abiotic stress in a diverse manner, emphasizing the importance of elucidating the different roles of *COMT* genes in different plant species. Additionally, a remaining question pertains to whether COMTs have a simultaneous impact on both lignin and melatonin biosynthesis.

*L. chuanxiong*, predominantly found in Chengdu plain, has been recognized as a significant edible-medicinal plant within *Apiaceae* family for centuries (Song et al., 2015). According to the 2020 edition of Chinese Pharmacopeia, *L. chuanxiong* is renowned for its synthesis of ferulic acid, a vital phenolic compound with diverse biological activities. Given the potential role of COMT in the ferulic acid biosynthetic pathway (Song et al., 2015), a *COMT* gene, denoted as *LcCOMT*, was cloned from *L. chuanxiong*, and its recombinant protein was demonstrated to catalyze the conversion of caffeic acid to ferulic acid for the first time (Li et al., 2015). Subsequent structural analysis revealed that LcCOMT exhibits a distinctive Rossmann-like fold crucial for its enzymatic function (Song et al., 2022). Recent studies have also highlighted *COMTs* in various species of *Apiaceae* family, such as *Angelica dahurica*, *Angelica glauca* Edgew, and *Saposhnikovia divaricata*, due to their potential involvement in the biosynthesis of downstream metabolites (Zhao et al., 2021; Devi et al., 2022; Kou et al., 2022), although direct verification is lacking. Therefore, investigating the role of *LcCOMT* gene in the accumulation of downstream metabolites and drought tolerance is of interest, as it may shed light on the biological function of COMTs in plants, particularly within *Apiaceae* family. This study firstly confirmed that LcCOMT significantly converts N-acetylserotonin into melatonin through bioinformatic analysis and *in vitro* enzymatic assays. Subsequently, the impact of *LcCOMT* gene on lignin and melatonin biosynthesis was explored, leading to the generation of *LcCOMT* transgenic *Arabidopsis* plants. Furthermore, the drought tolerance of these transgenic plants was evaluated, with potential applications for drought tolerance in the future. This study is one of the few to investigate the concurrent impact of *COMT* on lignin and melatonin biosynthesis. Furthermore, this is the first report on the *in vivo* biological function of *COMT* gene in a species of *Apiaceae* family.

## 2 Results

### 2.1 Sequence alignment and phylogeny of *LcCOMT*

Using ClustalW 2.1 and EsPript 3.0, an analysis was conducted on the sequence identities of LcCOMT in comparison to well-characterized ASMTs and COMTs associated with melatonin biosynthesis in diverse plant species (**Supplementary Table 1**) based on their amino acid sequences. LcCOMT demonstrated sequence identities of up to 41% with ASMTs from plant species such as *Arabidopsis*, *Malus zumi*, and *O. sativa*. Notably, it exhibited higher sequence identities with *COMTs* from *Solanum lycopersicum* (77%), *Gossypium hirsutum* (76%), and *Arabidopsis* (73%), indicating a closer biochemical resemblance to these COMTs.

**Figure 1:**
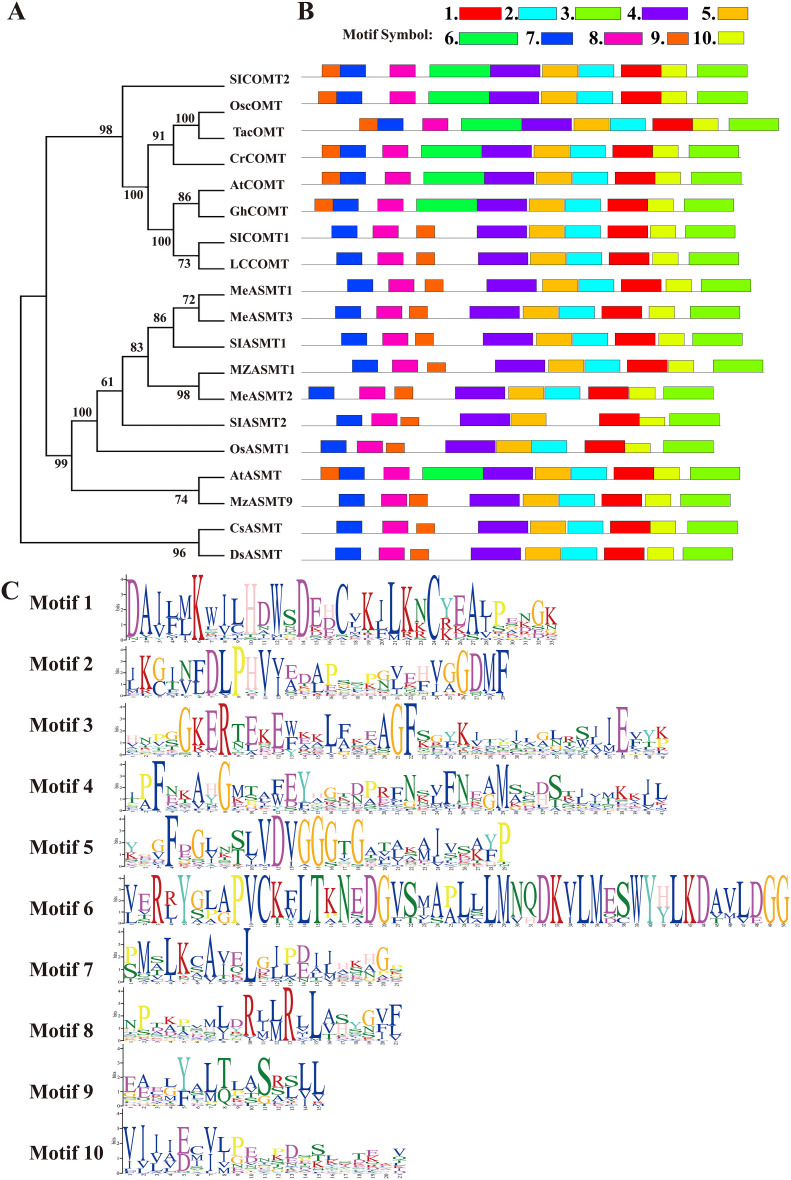
Phylogeny and conserved motif analysis of well-defined ASMT/COMTs and two bacterial ASMT proteins. **(A)** phylogeny; **(B)** motifs organization and **(C)** sequence logos of corresponding conserved motifs. At, *Arabidopsis thaliana*; Cl, *Citrullus lanatus*; Gh, *Gossypium hirsutum*; Lc, *Ligusiticum chuanxiong*; Me, *Manihot esculenta*; Mz, *Malus zumi*; Os, *Oryza sativa*; Sl, *Solanum lycopersicum*; Ta, *Triticum aestivum*; ASMT, N-acetylserotonin O-methyltransferase; COMT, caffeic acid O-methyltransferase.

Furthermore, the phylogenetic relationship between LcCOMT and established ASMT/COMTs was evaluated through a neighbor-joining (NJ) tree (**Figure 1A**). The high-support tree revealed distinct sister clades of COMTs and ASMTs, with over 56% and 94% of branches having bootstrap values exceeding 90 and 70, respectively (**Figure 1A**). Both COMTs and ASMTs formed monophyletic clades with robust bootstrap values of 98 and 99, indicating a strong association between amino acid similarity and COMT family relatedness. Notably, COMTs and ASMTs from the same species, such as *Arabiodpsis*, *O. sativa*, and *S. lycopersicum*, did not group together, suggesting that the differentiation between ASMTs and COMTs occurred prior to the speciation of these plants. LcCOMT, along with SlCOMT1, AtCOMT, and GhCOMT, known for their involvement in melatonin biosynthesis and melatonin-induced plant resistance (Li et al., 2019; Hasan et al., 2019), were clustered into a highly supported clade (100 BP).

Conserved motif patterns in COMTs were examined due to their shared O-methylation function in higher plants. Ten conserved motifs, labeled 1 to 10, were identified (**Figure 1B**). Generally, similar gene types contained identical motifs. Motifs 1, 3, 4, 5, 7, 8, 9, and 10 were present in all 19 members, with motifs 7 and 8 being associated with LcCOMT dimerization, and the others involved in substrate binding. Motif 6, which included *β*-2 sheet, *α*-6, *α*-7, and *α*-8 helices in LcCOMT (Song et al., 2022), was unique to COMTs, indicating a post-divergence development. Within motif 6, Leu126, Met129, and Asn130 may confer a novel function in lignin biosynthesis for COMTs. Notably, motif 9 exhibited positional variation between COMTs and ASMTs, being located at the N-terminus for COMTs and downstream for ASMTs. Comprising 15 amino acids in *α*-1 and *α*-2 helices, motif 9’s residues Cys19, Met20, and Ser26 (as numbered in LcCOMT) facilitated subunit interactions. Almost all proteins, with the exception of AFZ23489, contained motif 2. COMTs were found to possess three catalytic residues – His268, Asp269, and Glu328 – situated in motifs 1 and 3. Furthermore, motif 4, which spanned 41 amino acids and formed the longest α-11 helix, was conserved across all 19 members (**Figure 1C**).

**Figure 2:**
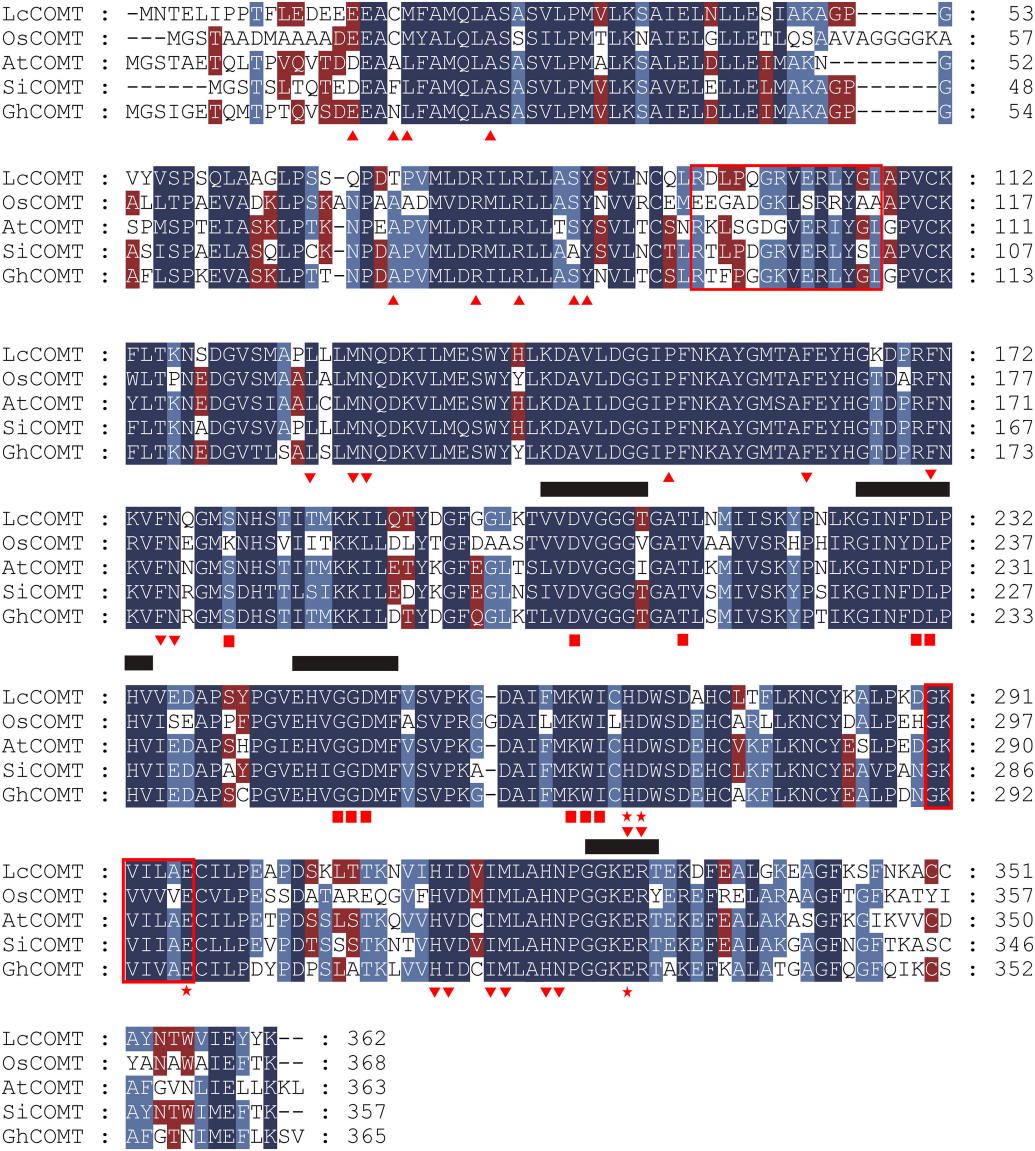
Sequence alignment of LcCOMT, OsCOMT, AtCOMT, SlCOMT1 and GhCOMT. The putative N-acetylserotonin-binding or serotonin-binding domains are shown in the red box. The residues in dimerization interface are indicated by regular triangle. The phenolic substrate-binding sites are marked with inverted triangle asterisks. The S-adenosyl-L-methionine (SAM)-binding sites are indicated as boxes. The catalytic residues are indicated by pentagram. The conserved function regions of COMTs, VVDTGGG(T/V/I)G, GIN(F/Y)DLPHV, EH(V/I)GGDMF, and GGKER(T/Y), are underlined.

Based on the crystal structures reported in the literature, COMTs are comprised of an N-terminal domain responsible for dimerization and a C-terminal domain involved in SAM and substrate binding (Song et al., 2022). The sequence alignment in **Figure 2** indicates a higher degree of conservation in residues that anchor both SAM and phenolic substrates among COMTs from diverse species, in comparison to those in the dimerization domain. Chang et al. (2021) identified two regions within *COMTs* that are involved in N-acetylserotonin binding, with region II exhibiting greater conservation than region I, where the residues Asp95 and Gln98 are specific to LcCOMT (**Figure 2**). Functional regions shared between ASMTs and COMTs exhibit varying levels of conservation, including VVDVGGG(T/V/I)G, GIN(F/Y)DLPHV, EH(V/I)GGDMF, and GGKER(T/Y) (Zhao et al., 2022b). Group-wise sequence alignment further corroborated the observation that COMTs demonstrated higher conservation than ASMTs across these four functional regions (**Figure 2**).

### 2.2 *In vitro* biochemical function of LcCOMT protein

**Figure 3:**
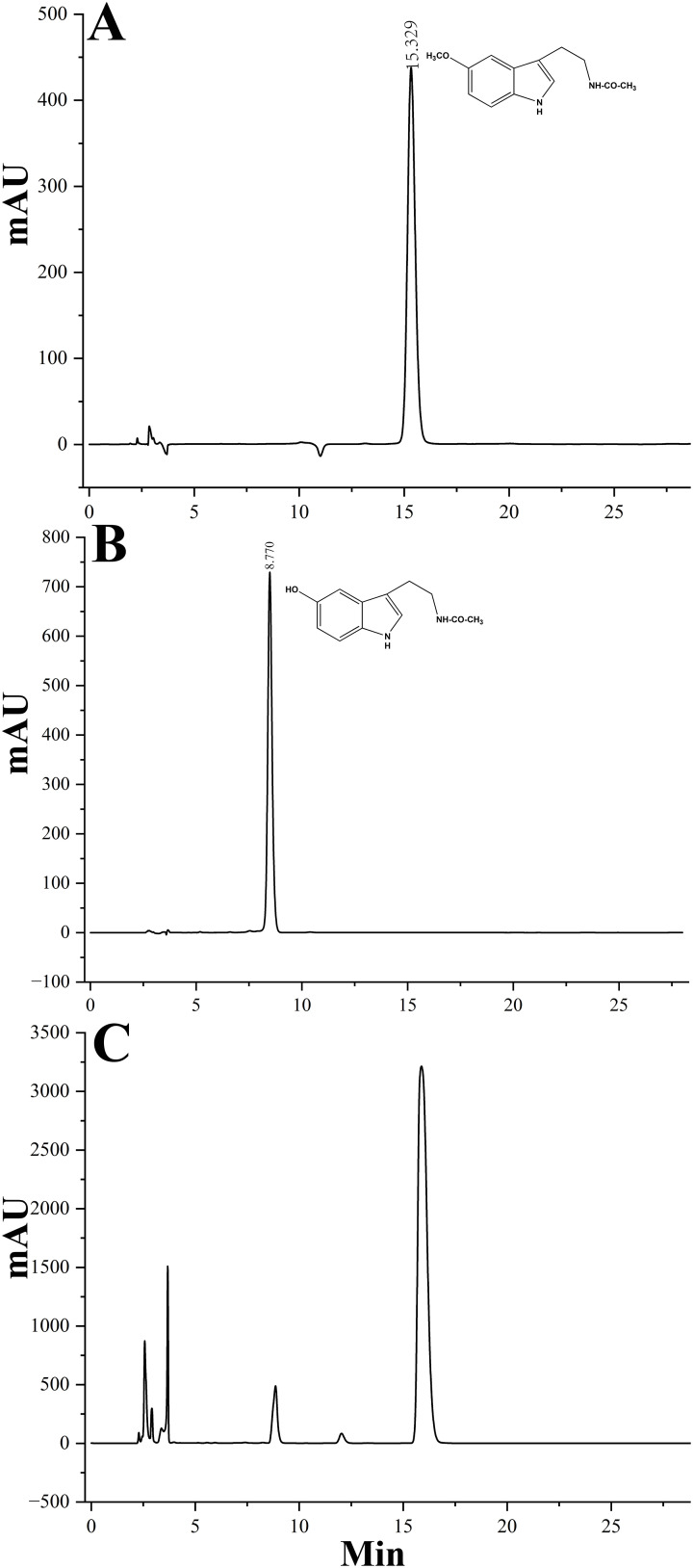
HPLC analysis of reaction products catalyzed by purified recombinant LcCOMT. **(A)** A control of melatonin, **(B)** a control of N-acetylserotonin, and **(C)** the reaction product of LcCOMT (N-acetylserotonin and SAM were added to the reaction mixture).

Given the high sequence identity often associated with shared biochemical functions, it is hypothesized that LcCOMT participates in the methylation reactions involved in both lignin and melatonin biosynthesis. His-tagged LcCOMT, obtained from *Escherichia coli*, was isolated through purification using a Ni-agarose column (**Supplementary Figure 1A**). Subsequent trypsin digestion followed by electrospray ionization tandem mass spectrometry (ESI-MS/MS) analysis revealed an 85% match with the theoretical sequence of LcCOMT (**Supplementary Figure 1B**), confirming the isolation of the protein in a pure form. Analysis in **Figure 3** and **Table 1** indicated that LcCOMT exhibited a notably higher affinity for caffeic acid compared to N-acetylserotonin, as indicated by their respective *K*m values (0.099 mM for caffeic acid and 0.328 mM for N-acetylserotonin, respectively) (**Table 1**). This preference aligns with observations in *COMTs* from *O. sativa* and *Arabiodpsis* (Back et al., 2016). Previous studies have reported COMTs with varying *K*m values for N-acetylserotonin in *N. tabacum* (0.266 mM, Yao et al., 2022) and *Arabidopsis* (0.74 mM, Wang et al., 2019), highlighting the diverse enzymatic characteristics of COMTs across different species. While LcCOMT exhibited a lower affinity (higher *Km*) for N-acetylserotonin compared to NtCOMT1, it displayed a higher *V*max than NtCOMT1 (2.155 nmol/min/mg protein). Instances of functional proteins with higher capacity but lower specificity have been documented in carboxylesterase (McCarthy and Witz, 1997), glucose transporter (Katagiri et al., 1992), and cation transporters (Jensen et al., 2020), primarily attributed to the flexibility within the enzyme’s active site microenvironment (Ahmed et al., 2020). The optimal temperature for N-acetylserotonin as a substrate was determined to be 37°C, suggesting the potential of LcCOMT as a candidate with high-temperature tolerance.

**Table 1:** Kinetic parameters of COMT proteins from various plants.

Species	Enzymes	*K*m (mM)	*V*max (nmol/min/mg)	Reference
*L. chuanxiong*	LcCOMT	0.328	7.994	
*Nicotiana tabacum*	NtCOMT	0.266	2.155	Yao et al., 2022
*Arabidopsis*	AtCOMT	0.233	1.8	Lee et al., 2014
*Arabidopsis*	AtCOMT	0.74	–	Wang et al., 2019

**Figure 4:**
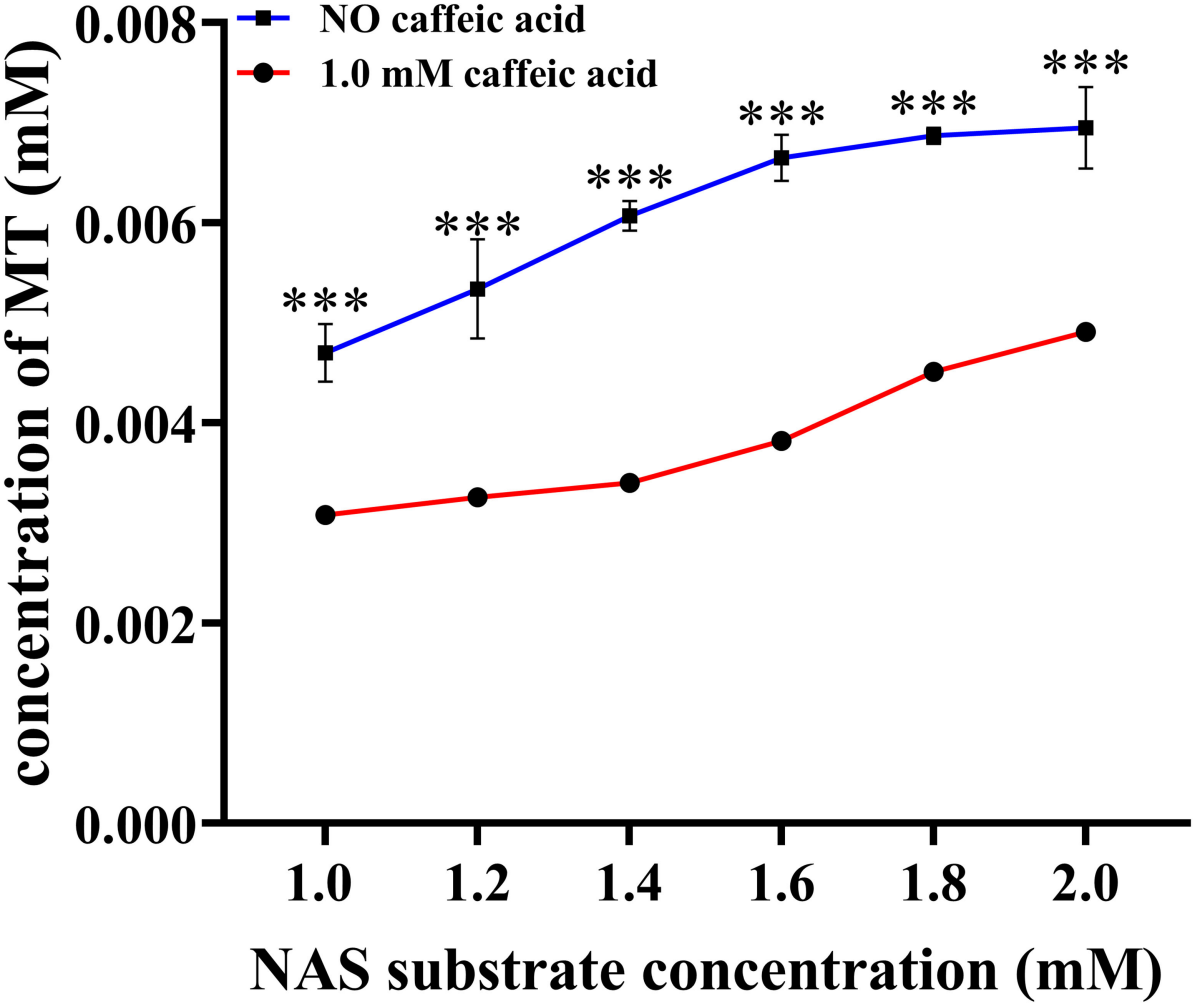
Caffeic acid inhibits the activity of LcCOMT against N-acetylserotonin. *** represented statistical significance of p<0.001.

Our previous molecular docking analysis suggested that Phe171 and Asp269 played crucial roles in the binding and catalytic processes of caffeic acid (Song et al., 2022). Phe171 in motif 4 exhibited substitutions with Leu, Ser, Ala, Met, Asp, and Val in various *ASMTs*, while Asp269 in motif 1 showed substitutions with Ala, Cys, Ileu, and Asn in different ASMTs (**Supplementary Figure 2**). To assess their significance, these residues were replaced with alanine, and the enzymatic activities of both site mutations were assessed. The results in **Supplementary Table 2** indicated that F171A and D269A mutations had different impacts on the catalytic properties of *LcCOMT*. The activity of LcCOMT-F171A towards caffeic acid decreased by 81.90%, whereas the reduction for N-acetylserotonin was only 10.86%, highlighting the importance of Phe171 in caffeic acid binding. In contrast, D269A mutation led to a 94.29% decrease in activity towards caffeic acid and only a 16.56% decrease for N-acetylserotonin, suggesting a lesser effect of Asp269 on N-acetylserotonin. Additionally, caffeic acid was found to inhibit the methylation of N-acetylserotonin; with 2.0 mM N-acetylserotonin and 1.0 mM caffeic acid, a 31.80% reduction in activity was observed (**Figure 4**).

### 2.3 Expression profile of *LcCOMT* gene in transgenic plants under drought treatment

**Figure 5:**
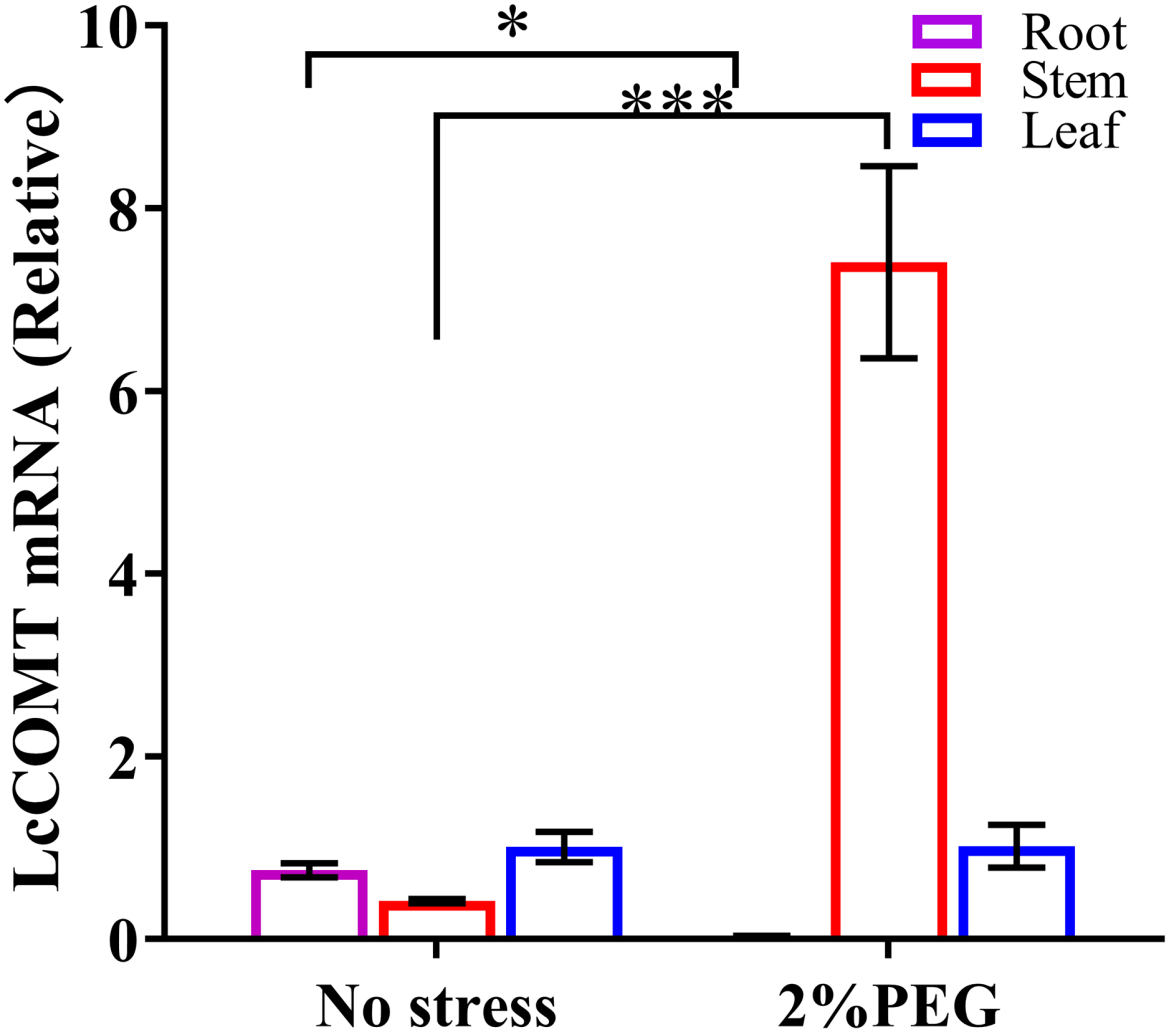
The expression of the *LcCOMT* gene in tissues of *LcCOMT* transgenic plants after drought treatments. * and *** represented statistical significance of p<0.05 and p<0.001, respectively.

Lignin and melatonin are recognized for their involvement in plant responses to abiotic and biotic stresses, with COMTs playing a crucial role in their biosynthetic pathways. In this study, leaf, stem, and root tissues were obtained from *LcCOMT* transgenic plants to investigate the expression patterns of *LcCOMT* gene following drought treatment (**Figure 5**).

Under normal conditions, there were no discernible differences in the expression of *LcCOMT* gene among the three tissues. However, under drought stress, the overall expression level of *LcCOMT* gene in these tissues significantly increased, reaching a 3.9-fold higher level compared to that under normal conditions. Particularly noteworthy is the observation that under drought stress, the expression level of *LcCOMT* gene in stem tissue exhibited the most substantial increase, showing an 18-fold higher expression level than that under normal conditions.

### 2.4 *LcCOMT* overexpression enhances transgenic *Arabidopsis* drought tolerance

**Figure 6:**
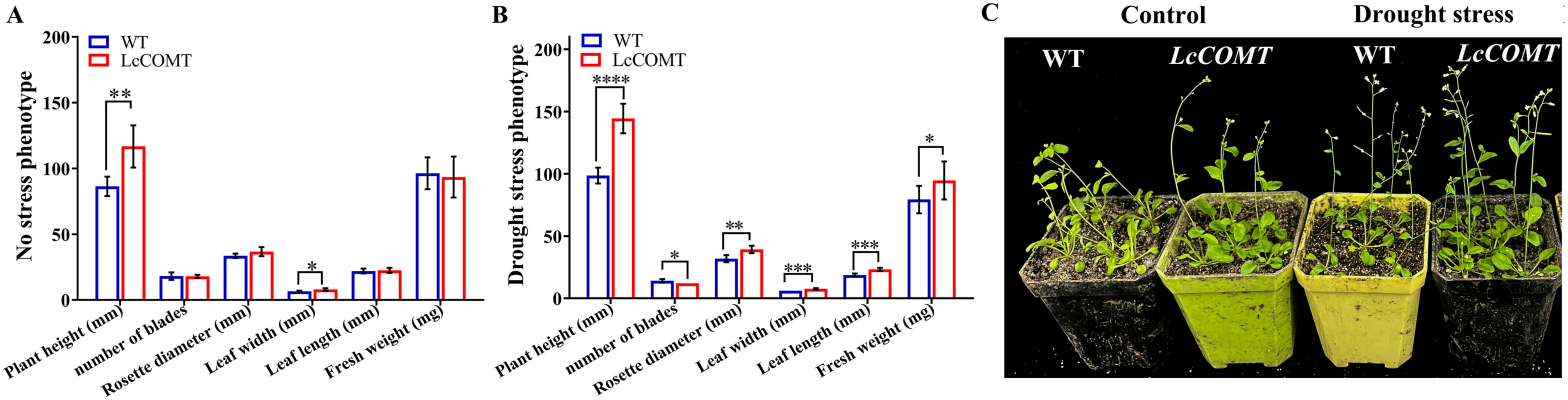
Phenotypic analysis of wild-type and *LcCOMT* transgenic plants. **(A)** No stress growth charts; **(B)** Drought stress growth charts; **(C)** No stress and drought stress phenotypic. WT, wild-type plants; LcCOMT, *LcCOMT* transgenic plants. *, **, *** and **** represented statistical significance of p<0.05, p<0.01, p<0.001 and p<0.0001, respectively.

Transgenic and wild-type *Arabidopsis* seeds were cultivated on 1/2 MS medium for 14 days, followed by 14 days of soil culture. Under normal conditions, there were no significant phenotypic variances between transgenic and wild-type plants, except for a slightly increased height, reduced leaf count, and lower fresh weight in the transgenic plants. The wild-type plants displayed decreased rosette leaf diameter, leaf length, leaf width, and fresh weight following drought treatment, potentially due to the elevated production of ROS induced by the drought stress (**Figure 6**). Remarkably, under drought stress conditions, transgenic plants demonstrated substantially greater plant height, leaf length, leaf width, and fresh weight, with statistical significance, in comparison to wild-type plants. Additionally, transgenic plants exhibited a larger rosette leaf diameter under drought stress. However, transgenic plants had slightly fewer leaves than wild-type plants under drought stress conditions (**Figure 6**). These results validated that the overexpression of *LcCOMT* gene significantly boosted tolerance to drought stress.

### 2.5 *LcCOMT* improves lignin and melatonin synthesis in transgenic plants under drought stress

**Figure 7:**
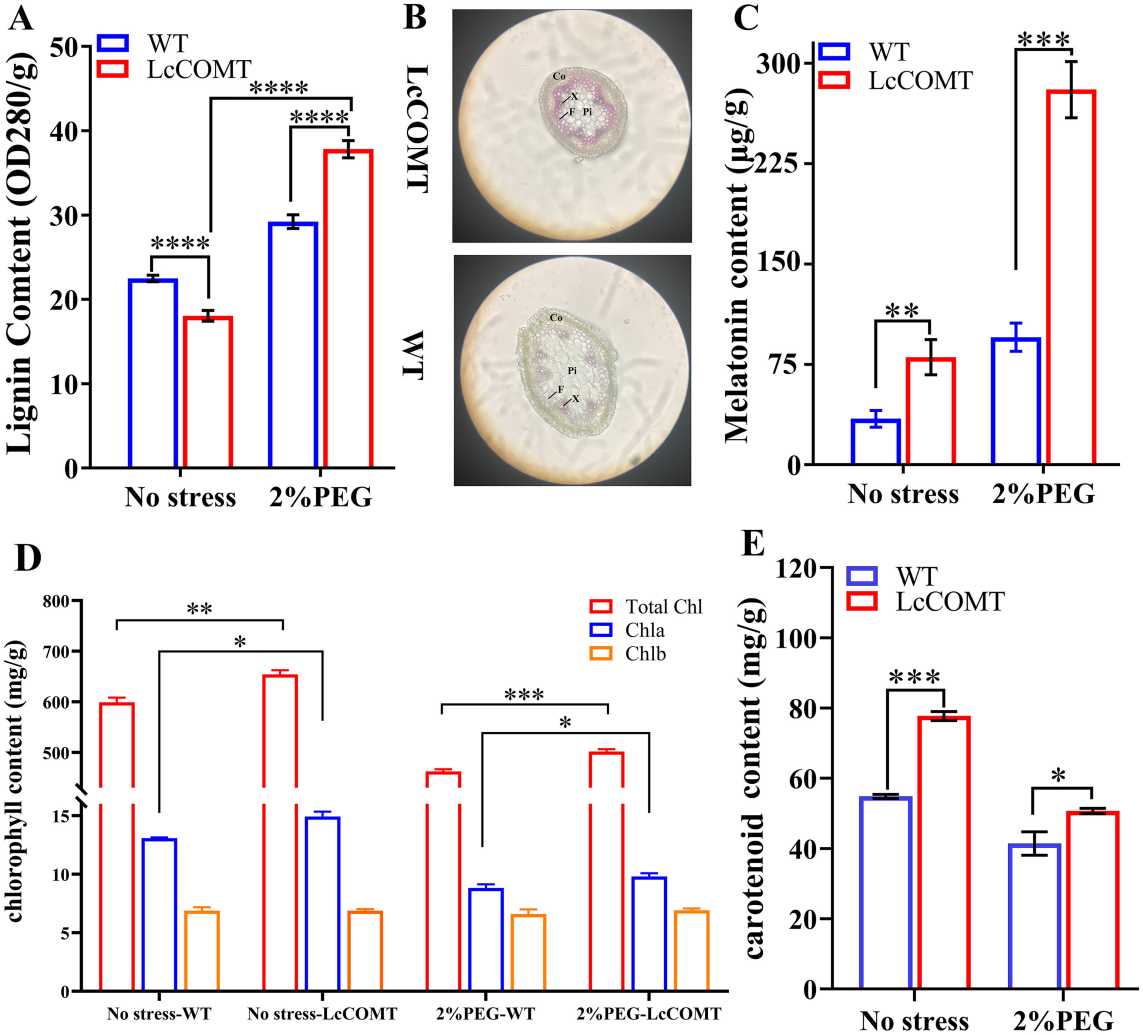
Photosynthetic pigment content, lignin and melatonin synthesis of wild-type and *LcCOMT* transgenic plants. **(A)** lignin content; **(B)** The visualization of stems in wild-type and *LcCOMT* transgenic plants. CO, F, Pi and X represent Cortex, vascular bundle, pith and xylem, respectively. The eyepiece was set as 10 folds; **(C)** melatonin content; **(D)** chlorophyll content; **(E)** *β*-carotene content. WT, wild-type plants; LcCOMT, *LcCOMT* transgenic plants. *, **, *** and **** represented statistical significance of p<0.05, p<0.01, p<0.001 and p<0.0001, respectively.

To assess the impact of modified *COMT* levels on downstream metabolites, the lignin and melatonin concentrations were evaluated in transgenic and wild-type plants. Under normal circumstances, the total lignin content in *LcCOMT* transgenic plants decreased to 80.3% of the levels found in wild-type plants (*p*<0.0001). However, following exposure to drought conditions, these transgenic plants demonstrated a 129.5% rise in lignin content compared to the wild-type plants (*p*<0.0001) (**Figure 7A**). Given that lignin is a key structural component of the cell wall and aids in the long-distance transportation of water and nutrients in higher terrestrial plants (Vanholme et al., 2019), the stem structures of wild-type and transgenic plants were compared post drought treatment. Consequently, the stems of *LcCOMT* transgenic plants accumulated more purplish-red lignin than those of wild-type plants. Additionally, the cortex in *LcCOMT* transgenic plants exhibited greater thickening compared to that in wild-type plants (**Figure 7B**), potentially offering enhanced protection in scenarios of water scarcity (Zhuang et al., 2020). Interestingly, under drought stress conditions, the transgenic plants displayed a more uniform pith structure in contrast to the wild-type plants, indicating that the transgenic plants might better withstand drought stress due to their improved capacity for water and nutrient transport compared to the wild-type plants (**Figure 7B**).

Overexpression of *LcCOMT* gene resulted in elevated melatonin production. In *LcCOMT* transgenic plants, the total melatonin content was 2.34 times higher compared to wild-type plants (*p*<0.001). Following drought treatment, this increase became more significant, with melatonin levels reaching 2.94 times that of the wild-type plants (**Figure 7C**). These results indicated that *LcCOMT* gene was involved in melatonin biosynthesis, potentially impacting the drought stress tolerance of transgenic plants.

Chlorophyll and carotenoids are pigments crucial for photosynthetic efficiency and response to stress (Gajardo et al., 2024). When exposed to drought stress, Chla/b ratio decreased compared to the control group. Specifically, Chla/b ratio in wild-type plants was approximately 1.90 under normal conditions, but decreased to 1.34 under drought stress, likely due to the adverse impacts of the stress. Interestingly, chlorophyll levels in *LcCOMT* transgenic plants were significantly higher at 108% (*p*<0.001) compared to wild-type plants (**Figures 7D, E**). Furthermore, Chla/b ratio in *LcCOMT* transgenic plants was 1.14 times higher than in wild-type plants under normal conditions, and 1.06 times higher under drought stress (**Figure 7D**). Additionally, carotenoid levels in *LcCOMT* transgenic plants were 122% (*p*<0.05) of those in wild-type plants (**Figures 7D, E**).

### 2.6 Overexpression of *LcCOMT* increases defense-related enzymes activities together with reduced MDA and H_2_O_2_ contents

Drought stress triggers the production of ROS such as H_2_O_2_ (hydrogen peroxide), leading to the oxidation of membrane lipids (Wilkinson and Davies, 2010; Shabala et al., 2015). Malondialdehyde (MDA) is widely recognized as a marker of the extent of membrane lipid oxidation (Huang et al., 2020). Antioxidant enzymes, such as superoxide dismutase (SOD), peroxidase (POD), and catalase (CAT), function as ROS scavengers, alleviating damage caused by ROS (Huang et al., 2020). To elucidate the drought-tolerant mechanism induced by *LcCOMT* overexpression, this study assessed the activities of POD, SOD, and CAT, observing enhanced activities in the transgenic plants.

**Figure 8:**
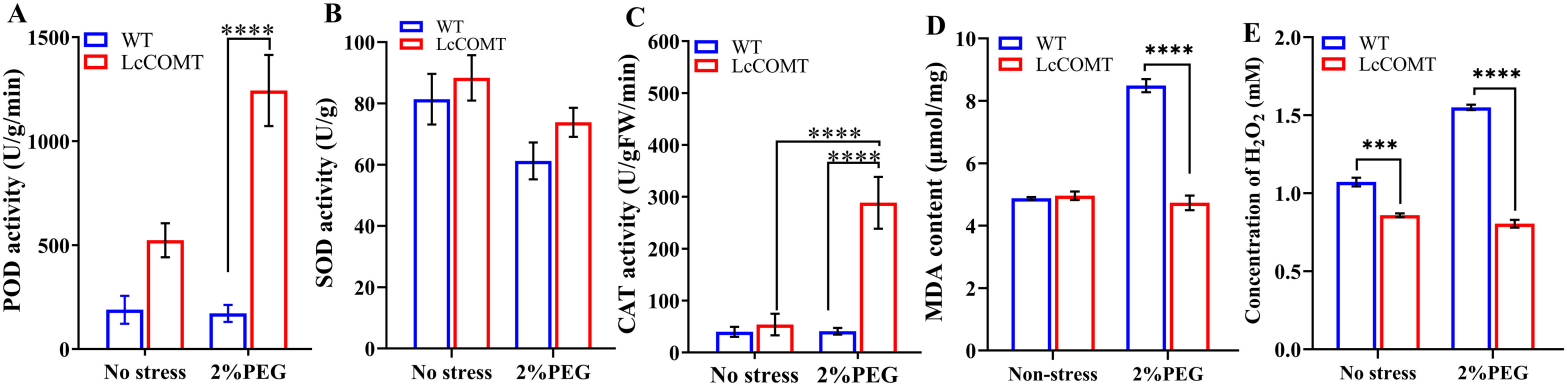
Anti-oxidant enzyme activities in wild-type and transgenic plants. **(A)** peroxidase (POD) activity; **(B)** superoxide dismutase (SOD) activity; **(C)** catalase (CAT) activity; **(D)** Malondialdehyde (MDA) content; **(E)** H_2_O_2_ content. WT, wild-type plants; LcCOMT, *LcCOMT* transgenic plants. *** and **** represented statistical significance of p<0.001 and p<0.0001, respectively.

**Figure 8A** demonstrated that POD activity was consistently elevated in transgenic plants compared to wild-type plants across various conditions, with the most notable increase observed after drought treatments. Specifically, the total POD activity in *LcCOMT* transgenic plants was 2.77 times higher than that in wild-type plants under normal conditions. Following exposure to drought stress, the POD activity in transgenic plants significantly rose to 1244.03 U/g/min, whereas it was only 172.00 U/g/min in wild-type plants (*p*<0.0001). In **Figure 8B**, SOD activity in transgenic plants (88.36 U/g) exceeded that in wild-type plants (81.40 U/g) under normal conditions. However, both transgenic and wild-type plants exhibited decreased SOD activity under drought stress compared to normal conditions. Furthermore, CAT activity in transgenic plants was 1.35 times higher than that in wild-type plants under normal conditions. Remarkably, under drought stress, CAT activity in transgenic plants was approximately 7 times higher than that in wild-type plants (**Figure 8C**), indicating that the overexpression of *LcCOMT* led to enhanced CAT activity.

**Figure 8D** revealed that there was no statistically significant variance in MDA content between transgenic and wild-type plants under normal conditions. However, MDA content in *LcCOMT* transgenic plants was observed to be lower than that in wild-type plants under drought stress. Additionally, H_2_O_2_ levels in *LcCOMT* transgenic plants were lower than those in wild-type plants under both normal conditions and drought stress (**Figure 8E**). These results suggested that the overexpression of *LcCOMT* enhanced the antioxidant capacity of transgenic plants, thereby improving their resilience to drought stress.

### 2.7 Phytohormone melatonin application increased defense-related enzymes activities

In contrast to lignin as a support material, previous studies have suggested that the phytohormone melatonin might enhance stress tolerance through its ability to scavenge ROS (Kolupaev et al., 2024). To explore the potential of melatonin in boosting the activities of defense-related enzymes, exogenous melatonin was administered to wild-type plants under both normal and drought conditions.

**Figure 9:**
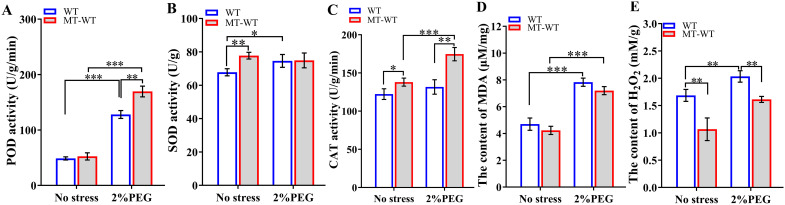
Defense-related enzyme activities after exogenous application of melatonin. **(A)** POD activity; **(B)** SOD activity; **(C)** CAT activity; **(D)** MDA content; **(E)** H_2_O_2_ content. WT, wild-type plants; MT-WT, wild-type plants treated by melatonin. *, **, and *** represented statistical significance of p<0.05, p<0.01, and p<0.001, respectively.

Biochemical assays conducted after 7 days of treatment revealed that the application of exogenous melatonin led to an increase in the activities of POD, SOD, and CAT enzymes (**Figure 9**). In comparison to untreated plants, melatonin-treated plants exhibited elevated levels of POD, SOD, and CAT enzymes, with SOD and CAT activities being 1.15 times (*p*<0.01) and 1.13 times (*p*<0.05) higher than those in the control group, respectively. Additionally, the levels of MDA and H_2_O_2_ were lower in melatonin-treated plants than in the control group, with H_2_O_2_ content decreasing to 63.26% (*p*<0.01) of the control levels.

Under conditions of drought stress, plants treated with melatonin demonstrated elevated activities of POD, SOD, and CAT in comparison to untreated plants. Specifically, the activities of POD and CAT showed a significant increase by 1.33-fold (*p*<0.01). Additionally, the application of melatonin resulted in decreased levels of MAD and H_2_O_2_, with H_2_O_2_ content decreasing to 79.27% (*p*<0.05) of the control levels. These results highlighted the essential role of melatonin in enhancing the activities of defense-related enzymes and suggested its potential involvement in promoting stress tolerance.

## 3 Discussion

COMTs, which are part of O-methyltransferase (OMT) family, play a crucial role in the methylation process involved in the biosynthesis of various metabolites, such as phenylpropanoids, flavonoids, alkaloids, and melatonin (Do et al., 2007; Besse et al., 2009). Melatonin has been identified in a wide range of organisms, from primitive bacteria to higher plants. The presence of COMTs in bryophytes raises questions about their evolutionary origins. The phylogenetic analysis might support the idea that angiosperm COMT/ASMTs have evolved from ancient ASMTs (**Figure 1A**), in line with the proposal by Zhao et al. (2022b) that COMTs evolved from ancestral ASMTs during the plant colonization of land. The four conserved domains shared by ASMT and COMT might serve as distinctive markers of OMT family (**Figure 2**). Furthermore, the phylogenetic analysis revealed that COMT/ASMTs from the same species, such as *O. sativa*, *Arabidopsis*, and *S. lycopersicum*, were located in separate evolutionary branches, suggesting that the genetic differences leading to these differences might have arisen before the divergence of these species. Notably, COMTs from *Arabidopsis* and *O. sativa* exhibited higher melatonin production compared to ASMTs, indicating that COMTs had not only preserved ASMT activity but also had significantly enhanced it (Zhao et al., 2022b). Additionally, COMTs have been recognized as key enzymes in lignin biosynthesis, primarily present in vascular tissues, providing terrestrial plants with tolerance to drought, salinity, and high temperatures (Qin et al., 2022; Rahman et al., 2022). To elucidate the biological properties of these enzymes, the research conducted an analysis of amino acid sequence variations among COMT/ASMTs, revealing substantial diversity, with LcCOMT sharing no more than 41% identity with ASMTs (**Supplementary Table 1**). Interestingly, a higher average pairwise identity was observed in COMTs (59%) compared to ASMTs (48%) from higher plants, indicating a greater conservation of COMTs following the divergence of COMT/ASMTs. The group-wise conserved motif analysis identified motif 6 as specific to COMTs, potentially contributing to the unique function of lignin biosynthesis (**Figures 1B, C**). In a previous study, it is confirmed that the residue Asn130 in motif 6 is associated with phenolic substrate binding (Song et al., 2022).

*In vitro* enzymatic assay demonstrated that LcCOMT effectively facilitated the conversion of N-acetylserotonin and caffeic acid. This substrate preference aligned with that of COMTs found in other plant species, despite notable kinetic variations among COMTs from different plants (Back et al., 2016). Tan et al. (2016) suggested that ASMTs from animals exhibited a stronger affinity for serotonin, underscoring the complex substrate specificity within COMTs. To investigate the mechanisms behind the different kinetic properties of LcCOMT, a comparison of N-acetylserotonin binding regions I and II in COMTs was conducted, revealing that binding region II is more conserved than region I. Specific residues such as Asp95 and Gln98, unique to binding region II of LcCOMT (**Figure 2**), might contribute to its kinetic differences, although further validation was required. Notably, while motif 1 and 4 were present in both COMTs and ASMTs, Phe171 in motif 4 and Asp269 in motif 1 were substituted by different amino acid residues in ASMTs, suggesting their potential involvement in substrate recognition (**Supplementary Figure 2**). Site mutation studies provided evidence that Phe171 and Asp269 played significant roles in the binding and catalysis of caffeic acid rather than N-acetylserotonin. The acetyl group in N-acetylserotonin could lead to changes in the binding position within the substrate pocket. Additionally, the production of melatonin by LcCOMT was hindered by caffeic acid, indicating a notable reduction in methylating activity. Lee et al. (2014) observed that caffeic acid inhibited the methylation of serotonin catalyzed by AtCOMT, implying shared binding sites for various substrates, including caffeic acid and N-acetylserotonin. Future studies should comprehensively identify the amino acid residues involved in substrate binding, particularly those interacting with N-acetylserotonin.

Recent omics studies suggested that COMT might not only function as stress-responsive proteins in stress regulatory networks but also play a role in plant tissue development (Khakdan et al., 2017; Liao et al., 2023; Pham et al., 2024). COMTs exhibit complex expression patterns in various tissues. For example, eight out of 18 *COMTs* from mango were down-regulated in roots, while seven out of those were upregulated in stems (Wang et al., 2024). However, 12 out of 16 *COMTs* from watermelon were predominantly up-regulated in roots (Chang et al., 2021). The quantitative real-time polymerase chain reaction (qRT-PCR) data indicated that *LcCOMT* in transgenic plants was not tissue-specific under normal conditions but showed significant upregulation in stems under drought stress, suggesting its potential role in the transport of mineral salts, nutrients, and water (**Figure 5**). A similar trend was observed with the upregulation of Bt toxin expression in Bt transgenic *O. sativa* under various environmental conditions, such as changes in CO_2_ concentration (Jiang et al., 2017). Comparable drought-induced expression levels of COMT proteins and/or transcripts have been documented in *N. tabacum* (Yao et al., 2022) and *Citrullus lanatus* (Chang et al., 2021). The expression pattern of *LcCOMT* in transgenic plants under drought stress aligned with that in its native plant, *L. chuanxiong*, where *LcCOMT* was induced under drought stress (Li et al., 2015). Given the stress-induced characteristics of *LcCOMT* gene in transgenic plants, it was plausible to suggest that *LcCOMT* gene could be a significant stress-responsive contributor.

To investigate the stress tolerance potential, a comparison was made between the adaptability and growth of transgenic and wild-type plants under normal and drought conditions. The results indicated that under drought stress, transgenic *Arabidopsis* exhibited improved adaptability and growth in comparison to wild-type plants (**Figure 6**). Similar positive results were observed in functional investigations of *COMTs* from other plant species, such as *C. rigescens* (Zhang et al., 2019), *C. lanatus* (Chang et al., 2021), and *O. sativa* (Choi et al., 2017). These results suggested that *LcCOMT* might have a positive impact on the drought response in *Arabidopsis*, thereby enhancing the comprehension of complex systems in higher plants, particularly in relation to drought tolerance. The genetic modifications based on *LcCOMT* gene showed promise for the development of stress-tolerant plants in the future. Plant responses to osmotic stresses such as drought are regulated through both abscisic acid (ABA)-dependent and ABA-independent signaling pathways (Yoshida et al., 2015). The identification of ABA response elements in the promoter of *COMT* gene, which exhibits high expression levels under ABA treatment (Zhang et al., 2019), suggests that while *COMT* is not directly involved in ABA biosynthesis, the improved drought tolerance observed in transgenic plants overexpressing *COMT* gene is likely associated with ABA-dependent pathways.

Lignin, a byproduct of the phenylpropanoid pathway, is acknowledged not only as a crucial element for providing mechanical support in plants but also for alleviating the impacts of abiotic stresses. The process of lignification predominantly takes place in various vascular tissues, contributing to the enhancement of plant height, stem strength, and yield (Kamran et al., 2018). Despite *LcCOMT* transgenic plants displaying reduced lignin levels compared to wild-type plants under normal circumstances, the heightened lignin levels in transgenic plants under drought stress potentially played a role in their improved growth during drought conditions (**Figures 7A, B**). Previous research has demonstrated that increased lignin levels can boost plant resilience to drought (Chen et al., 2020; Xu et al., 2020; Qin et al., 2022), suggesting that the lignin levels in *LcCOMT* transgenic plants could enhance drought tolerance. Additionally, while COMTs serve as pivotal enzymes in phenylpropanoid metabolism and their overexpression can elevate lignin content, the impact of heightened melatonin levels in these transgenic plants on enhancing lignin content should also be taken into account. Studies have indicated that the external application of melatonin could affect the expression of phenylpropanoid biosynthesis genes in cotton (Li et al., 2019), implying that the observed variations in lignin levels (**Figures 7A, B**) might be partly attributed to altered melatonin content.

Melatonin, a classical phytohormone, plays a beneficial role in modulating plant responses to both biotic and abiotic stress (Shi et al., 2015; Ge et al., 2023). The accumulation of melatonin and lignin has been observed to lead to various phenotypic changes that help plants to cope with stress (Huang et al., 2024). The biosynthesis of melatonin in plants involves two pathways originating from serotonin (Li et al., 2022a). The first pathway entails the conversion of serotonin to *N-acetylserotonin* by *serotonin N-acetyltransferase* (*SNAT*), followed by methylation through *ASMT* or *COMT* to produce melatonin. Conversely, the second pathway involves the initial methylation of serotonin into 5-methoxytryptamine by COMT or ASMT, followed by N-acetylation by SNAT to form melatonin (Choi et al., 2017). Considering that COMTs exhibit higher affinity and reaction efficiency towards N-acetylserotonin than serotonin, it was reasonable to assume that the first pathway played a more crucial role in melatonin biosynthesis in plants compared to the second pathway (Back et al., 2016). The significant enzymatic conversion of LcCOMT protein to produce melatonin suggests that enhancing melatonin levels in *LcCOMT*-overexpressing plants holds promise. Consequently, melatonin levels in *LcCOMT* transgenic plants were found to be higher under both normal and stress conditions compared to wild-type plants (**Figure 7C**). This increase in melatonin could potentially enhance plant tolerance to drought (Tiwari et al., 2021), indicating that stress tolerance regulation by *LcCOMT* may be linked to melatonin levels. The diverse functions of COMT (Li et al., 2016), underscore the significant role of *B. napus COMT* in various physiological processes. Several mechanisms have been proposed to explain the multifunctionality of melatonin in enhancing resistance to various abiotic stresses, including (1) induction of endogenous plant hormones and their related genes under stress (Arnao and Hernández-Ruiz, 2018); (2) protection of phytonutrients such as carotenoids, chlorophylls, flavonoids, and fibers (Sun et al., 2020; Arnao and Hernández-Ruiz, 2021); (3) enhancement of defense enzyme activities and production of antioxidative substances (Wang et al., 2021). In this study, *LcCOMT* transgenic plants exhibited higher chlorophyll and carotenoid contents, increased activities of POD, CAT, and SOD, and lower ROS content, potentially induced by drought stress (Hessini et al., 2021). Chlorophyll and carotenoids are essential components in photosystem I and II (PSI and PSII), aiding in light capture and transfer (Luimstra et al., 2019). The relatively higher chlorophyll content, Chla/Chlb ratio, and carotenoid content in *LcCOMT* transgenic plants compared to wild-type plants (**Figure 7D**) might enhance the conversion of light energy into biochemical energy, indirectly reducing ROS (Ren et al., 2023). Furthermore, the exogenous application of melatonin confirmed its role in promoting antioxidant enzyme activities while reducing MDA and H_2_O_2_ contents (**Figure 9**). Overall, the increased melatonin content in transgenic *Arabidopsis* under drought stress emphasized the significance of *LcCOMT* gene in melatonin biosynthesis. Changes in melatonin content might contribute to the adaptive variations associated with stress, with accumulated melatonin providing multiple positive effects on plant stress tolerance.

Furthermore, while *LcCOMT* enhances drought tolerance in plants through its pleiotropic effects, several aspects of its anti-stress mechanism require further discussion. Firstly, melatonin affects the biosynthesis of phenylpropanoids such as ferulic acid, *p*-coumaric acid, and *p*-hydroxybenzoic acid, which serve as precursors of lignin (Yin et al., 2022). Therefore, the alterations in lignin content (**Figures 7A, B**) may be partially attributed to changes in melatonin levels. Secondly, there is limited research on the regulation of melatonin biosynthesis, and certain key regulatory points remain unclear. For example, transgenic *O. sativa* overexpressing *O. sativa SNAT* exhibited low melatonin content despite high enzymatic activity (Byeon et al., 2015). Therefore, an evaluation of the impact on the genes related to melatonin biosynthesis in *LcCOMT* transgenic plants is warranted. Thirdly, considering the inhibitory effect of caffeic acid on N-acetylserotonin, the balance between lignin and melatonin biosynthesis should not be overlooked, and the metabolic flux may partly depend on the substrate concentrations. Fourthly, despite COMT evolving from ancient *ASMT* with a new function in lignin biosynthesis (Zhao et al., 2022b), their functional characteristics remain ambiguous. For instance, recombinant ASMT proteins were found to be inactive in catalytic analysis, but transgenic *O. sativa* overexpressing *ASMT* gene exhibited increased ASMT levels compared to wild-type plants (Park et al., 2013). Wang and Pichersky (1999) reported that despite eugenol/isoeugenol OMT and COMT sharing 83% amino acid sequence identity, they displayed different substrate preferences and methylation site specificities due to a few amino acid variations. In this study, LcCOMT demonstrated higher *K*m and *V*max parameters for N-acetylserotonin, despite sharing over 70% identity with *O. sativa* and *Arabiposis COMTs*. It is hypothesized that key amino acid substitutions may induce conformational changes in the substrate-binding region, resulting in kinetic differences. However, the selective and catalytic mechanism of COMTs remains partially unresolved due to the absence of three-dimensional structures of COMT/N-acetylserotonin complexes. Fifthly, as the biosynthetic sites of melatonin are linked to multiple organelles, including chloroplasts, mitochondria, endoplasmic reticulum, cytoplasm, and nucleus (Back et al., 2016; Tan and Reiter, 2020; Yao et al., 2022), it is imperative to provide more subcellular information on COMTs for targeted metabolic engineering of melatonin.

## 4 Conclusion and future direction

The bioinformatic analysis suggested an association between LcCOMT and the biosynthesis of melatonin and lignin. Subsequently, it is demonstrated that LcCOMT is capable of producing melatonin through O-methylation of N-acetylserotonin, despite the higher preference for caffeic acid, a precursor in lignin synthesis. The residues Phe171 and Asp269 in LcCOMT were found to primarily participate in the O-methylation process of caffeic acid rather than N-acetylserotonin. Through transgenic experiments, *LcCOMT* gene was confirmed to have a positive impact on drought tolerance. Furthermore, the enhanced accumulation of lignin and melatonin contents was identified as a potential molecular mechanism contributing to the drought tolerance mediated by LcCOMT. In conclusion, this study has contributed to a deeper understanding of the drought tolerance mechanisms in the plant COMT family, particularly through the mediation of lignin and melatonin biosynthesis.

Future research endeavors should delve into a more comprehensive investigation of various aspects encompassing morphology, physiology, and molecular mechanisms. Firstly, apart from aerial tissues, subterranean tissues will be utilized to assess the drought tolerance of *LcCOMT* transgenic plants. Secondly, meticulous examination of the subtle morphological traits of the vascular system and stomatal structures will be conducted. Thirdly, a detailed evaluation of several physiological characteristics, such as the PSI/PSII ratio, will be undertaken to enhance comprehension of the role of photosynthetic capacity in drought tolerance. Fourthly, transgenic plants harboring mutated *LcCOMT* genes will be engineered, followed by an assessment of their drought tolerance. Fifthly, an integrated omics approach will be employed to explore the downstream genes and metabolites involved in the melatonin-dependent signaling pathway, thereby potentially elucidating the molecular mechanisms of *LcCOMT* gene in drought tolerance. Sixthly, urgent measures include protein crystallization or the development of a homology model of LcCOMT (mutant LcCOMT)/N-acetylserotonin complex to unravel the catalytic mechanism of LcCOMT utilizing N-acetylserotonin as a substrate. Finally, determining the subcellular localization of LcCOMT is essential.

## 5 Materials and methods

### 5.1 Plants, chemicals, enzymes, plasmids, and bacterial strains

Briefly, the recombinant vector pET28a-*LcCOMT* is constructed by restriction enzyme digestion and ligation based on our previous research (Song et al., 2022). Trizol Reagent, Plant RNA Kit, PrimeScript™ RT reagent kit with gDNA Eraser (Perfect Real Time), size markers for DNA and proteins, SYBR Premix Ex Taq™ II, and restriction endonucleases were sourced from TaKaRa Bio and Tsingke Biotechnology. The Plasmid Mini Kit I was acquired from Vazyme Biotech. *Escherichia coli* (*E*. *coli*) strains DH5α™ and Agrobacterium GV3101 were provided by Invitrogen.

### 5.2 Sequence comparison, phylogeny and motif analysis of LcCOMT with other ASMT/COMTs related to melatonin biosynthesis

The nucleotide sequences of several functionally identified ASMT/COMTs, including nine ASMTs, eight COMTs, and two bacterial ASMTs as outgroups, were downloaded from GenBank database in NCBI (https://www.ncbi.nlm.nih.gov/) and were listed in **Supplementary Table 1**. Then, sequence alignments of selected full-length ASMT/COMT proteins were performed using ClustalW with default settings. A phylogenetic tree was constructed via the neighbor-joining (NJ) method with 1000 bootstrap replicates in MEGA 6.0 software (Zhang et al., 2021). MEME Suite (https://meme-suite.org/meme/tools/meme) was conducted to discover sequential motifs that represented structural features of ASMT/COMTs (Tian et al., 2023).

### 5.3 Expression and purification of LcCOMT protein in *E. coli*

Following the method outlined by Song et al. (2022), *E. coli* BL21 (DE3) harboring the pET28a-*LcCOMT* plasmid was cultured at 37°C in 500 mL of LB medium containing 0.1 mg/mL kanamycin. After the absorbance at 600 nm (OD600) of the bacterial solution reached approximately 0.6-0.8, 1 mM IPTG was added to induce production of LCCOMT protein for 16 hours. The bacterial cells were then harvested by centrifugation at 4°C and 5000×*g* for 10 min. The pellet was lysed in 20 mM Tris/HCl (pH 7.9) containing 0.1% (W/V) lysozyme and 150 mM NaCl, followed by sonication. The lysate was centrifuged at 4°C and 13,000×*g* for 10 minutes to collect the supernatant. Following the addition of supernatant to a Ni-agarose affinity column, the LcCOMT protein was eluted according to the manufacturer’s instructions (Takara, Japan). The purified LcCOMT protein was dialyzed in buffer composed of 2 mM Tris-HCl (pH 7.4), 10% (V/V) glycerol, 0.5 mM *β*-mercaptoethanol and 200 mM NaCl, and stored at -20°C. The commercial kit (BL521A, Biosharp Cor, China) developed from the Braford method was used to assess the concentration of the purified LcCOMT protein. The purity of the LcCOMT protein was assessed using 12.5% SDS-PAGE followed by Coomassie Blue staining (Li et al., 2015).

### 5.4 Enzymatic assay of LcCOMT protein

To determine the activity of LcCOMT against caffeic acid, we followed the procedure outlined in a previous report (Lee et al., 2014; Li et al., 2015) with minor modification. In brief, the enzymatic system consisted of 10 μg/mL purified LcCOMT protein, 200 μM substrate (caffeic acid or N-acetylserotonin), 1 mM SAM, 1 mM DTT and 100 μM potassium phosphate buffer (pH 7.8). After incubation at 37°C for 30 min, the reaction was terminated by adding 1 mL of methanol. The product was extracted three times by ethyl acetate and was then analyzed by high performance liquid chromatography (HPLC). In order to obtain the kinetic characteristics, the Lineweaver-Burk plots were generated with different concentrations of substrates to measure substrate affinity (*K*_m_) and maximum reaction rate (*V*_max_).

### 5.5 Site-directed mutation of LcCOMT protein

Phe171 and Asp269 were predicted as potential catalytic residues of LcCOMT protein via molecular docking (Song et al., 2022). To identify the speculation, site mutagenesis was performed using a point mutation kit (Vazyme, China) (Zhou et al., 2020). The codons TTT (Phe171) and GAT (Asp269) were altered to GCT (Ala) using the primer pairs LcCOMT-F171A-F, LcCOMT-F171A-R, LcCOMT-D269A-F and LcCOMT-D269-R (**Supplementary Table 3**), respectively. The PCR was performed by based on in 50 μL reaction volume containing 1 μL of Phanta Max Super-Fidelity DNA Polymerase, 1 μL of pET28a-*LcCOMT* as the template, 2 μL of forward primer, 2 μL of reverse primer, 1 μL of dNTP mix, 25 μL of 2×Max buffer, and 18 μL of sterile distilled water. The PCR product was digested by Dpn I and was then ligated into the linearized pET28a vector to produce mutants pET28a-*LcCOMT* D269A and pET28a-*LcCOMT* F171A. The bacterial transformation, expression, purification, and enzymatic assays of the mutated protein were performed using the same methods as for pET28a-*LcCOMT*.

### 5.6 Production of *LcCOMT* transgenic *Arabidopsis* plants

The recombinant vector carrying the *LcCOMT* gene was constructed using homologous recombination method (Yan et al., 2011). Initially, *LcCOMT* cDNA was amplified using the forward primer LcCOMT-KpnI-F and the reverse primer LcCOMT-KpnI-R, both containing the KpnI site (**Supplementary Table 3**). The *LcCOMT* fragment and vector pCambia2301-KY underwent linearization by KpnI digestion, following by purification and final conjugation to generate recombinant pCambia2301-KY-*LcCOMT* vector. The recombinant vectors were verified using the primers 35S-F and LcCOMT-R to confirm the correct orientation of the open reading frame. The transformation of *Arabidopsis thaliana* was performed via the Agrobacterium-mediated flower dip method as reported by Qin et al. (2022).

### 5.7 Drought treatments

The seeds of transgenic and wild-type plants were firstly germinated for 14 days on sterile half-strength Murashige and Skoog (MS) solid medium at 25°C, 80% humidity, and a 16 h light/8 h dark cycle. Seedlings were then transferred to soil pots (nutrient soil: vermiculite: peat, 1.5:1:1, V/V/V) and grown under the same light and temperature conditions. For drought treatment, seedlings were supplemented with 2% PEG (V/V). Control plants were sprayed with Holland nutrient solution. After 14 days, phenotypes of both transgenic and wild-type plants were evaluated.

### 5.8 Expression pattern of *LcCOMT* in transgenic plants under drought treatment

To analyze *LcCOMT* expression levels in transgenic plants under drought stress, qRT-PCR was performed as previously described (Li et al., 2015). Total RNA was extracted using the RNA simple Total RNA Kit (TIANGEN, Beijing, China), followed by reverse transcription using the FastKing RT Kit (TIANGEN, Beijing, China). qRT-PCR assays were conducted with the. Real-time PCR was performed in a 16 μl reaction volume containing 1 μl of template DNA, 1 μL of forward primer, 1 μL of reverse primer, 8 μL of SYBR^®^ Premix Ex Taq™ II (2×) kit (Takara, Japan), and 5 μL of sterile distilled water. Each run underwent one cycle at 94°C for 60 s, followed by 40 cycles at 94°C for 10 s, 60°C for 10 s, and 72°C for 20 s. Relative mRNA expression was quantified by normalizing to *Arabidopsis* 18S rRNA, calculated using the ΔΔCT-method (Li et al., 2015). The gene-specific primers (LcCOMTqFP, LcCOMTqRP, 18SFP, and 18SRP) are listed in **Supplementary Table 3**.

### 5.9 Determination of lignin, melatonin and chlorophyll content

Plant stems were powdered in liquid nitrogen and 10 mg of the freeze-dried powder was incubated in 2 mL of acetyl bromide/acetic acid (1:3, V/V) at 70°C for 30 minutes. The reaction was terminated by adding 0.9 mL of 2 M NaOH, 3 mL of acetic acid, and 0.1 mL of 7.5 M hydroxylamine hydrochloric acid. The acetyl bromide-soluble lignin content was determined following the same method as Qin et al. (2022). Meanwhile, the lignin was visualized using phloroglucinol-HCl staining as described by Qin et al. (2022). The stems located at 3 cm high of rosette leaves from transgenic and wild-type plants were soaked in FAA fixing solution for 3 weeks. The lignin in stems was stained by Wiesner color reaction, and thus the lignin-stained stems were observed immediately under the range of binocular optical microscope.

Melatonin content was quantified using the HPLC method (Martins et al., 2017). Initially, 0.2 g of stem sample was ground in liquid nitrogen and was extracted overnight in 1 mL of methanol. After centrifugation at 10,000 rpm, 4°C for 10 minutes, the supernatant was dissolved in 0.5 mL of 80% methanol and was filtered through a 0.45 μm membrane. The melatonin sample was then analyzed by HPLC at a flow rate of 1 mL/min with a mobile phase of acetonitrile/0.1% acetic acid (15:85, V/V).

Chlorophyll and carotenoid content were determined by optical absorbance measurement (Upreti et al., 2021), with minor modifications. Initially, 0.1 g of plant sample was ground with ice-cold 95% ethanol. The powder sample was kept in the dark for 12 hours at room temperature until the absorbance of the extract at 470, 649, and 665 nm was recorded, respectively. Total chlorophyll and carotenoid were calculated using the formulas: Chlorophyll a (C*a*)=13.95A*_665_*-6.88A*_649_*, chlorophyll b (C*b*)=24.96A*_649_*-7.32A*_665_*, Total chlorophyll=C*_a_* +C*_b_*; Carotenoid=(1000A*_470_*-2.05C*a*-114C*b*)/245.

### 5.10 Assays of POD, CAT and SOD activities

Leaf samples (500 mg fresh weight) were ground in liquid nitrogen. The powder was homogenized in 6 mL of 50 mM ice-cold sodium phosphate buffer (pH 7.8) for preparation of SOD samples. For preparation of POD and CAT samples, the buffer was changed by 50 mM sodium phosphate buffer (pH 7.0). The homogenates were centrifuged at 4°C, 10,000 rpm for 20 minutes, and the supernatant (crude extract) was used for enzyme assays.

CAT activity was determined using a commercial CAT assay kit (R22073-100T, Yuanye Cor, China) coupled with absorbance detection at 240 nm. CAT activity was monitored by the decrease in absorbance at 240 nm due to H_2_O_2_ decomposition, and defined as a 0.01 absorbance change per minute per mg of protein (Grilo et al., 2020). POD activity was measured by guaiacol oxidation and defined as an increase in absorbance at 470 nm using a Thermo Multiskan spectrometer (Shah and Nahakpam, 2012). SOD activity was assessed using a commercial SOD assay kit (ab65354, Abcam) according to the manufacturer’s instructions, evaluating its ability to inhibit the photo-reduction of nitro blue tetrazolium (NBT) (Tian et al., 2023).

### 5.11 Assays of malondialdehyde and hydrogen peroxide contents

By use of the commercial MDA kit (R21869, Yuanye Cor, China), the sample was prepared according to the instruments. The MDA content was determined based on its reaction with thiobarbituric acid (TBA), producing a pink pigment (Wang et al., 2018). Then absorbance of the pigment was recorded at 450, 532, and 600 nm. Total MDA content was calculated using the formulae. MDA=6.45(A*_532_*-A*_600_*)-0.56A*_450_*.

For preparation of sample for analysis of H_2_O_2_ content, 2 g fresh leaves were homogenized in 2 mL of ice-cold acetone followed by centrifugation at 12000×g, 4°C for 20 min. H_2_O_2_ content was quantified using a commercial TiO_2_ kit (R30339-50T, Yuanye Cor, China). TiO_2_ reacts with H_2_O_2_ to produce H_2_O_2_/TiO_2_ complex, a yellow compound with an absorption maximum at 412 nm.

### 5.12 Statistical analysis

Data from this study underwent statistical analyses utilizing SPSS v19.00 (SPSS Inc., United States). The results are expressed as the mean ± standard deviation of three values (N=3). To ascertain statistically significant differences between two groups, independent *t*-tests were employed and those with *p*-values less than 0.05 were deemed statistically significant. For *K*m and *V*max assessment, three replicates were set up in each group, and one-way ANOVA test was performed using the curve fitting function of GraphPad Prism 9.5.0 with a threshold of 0.05.

## Data Availability

The original contributions presented in the study are included in the article/**Supplementary Material**, Further inquiries can be directed to the corresponding authors.
