# Supplementary material for: Caffeic acid O-methyltransferase from Ligusticum chuanxiong alleviates drought stress, and improves lignin and melatonin biosynthesis
Source: Front Plant Sci. 2024 Sep 18;15:1458296. doi: 10.3389/fpls.2024.1458296 (PMC11445181; doi:10.3389/fpls.2024.1458296)
Supplement: Supplementary file 1 [file DataSheet1.docx]

**Supplementary Materials**


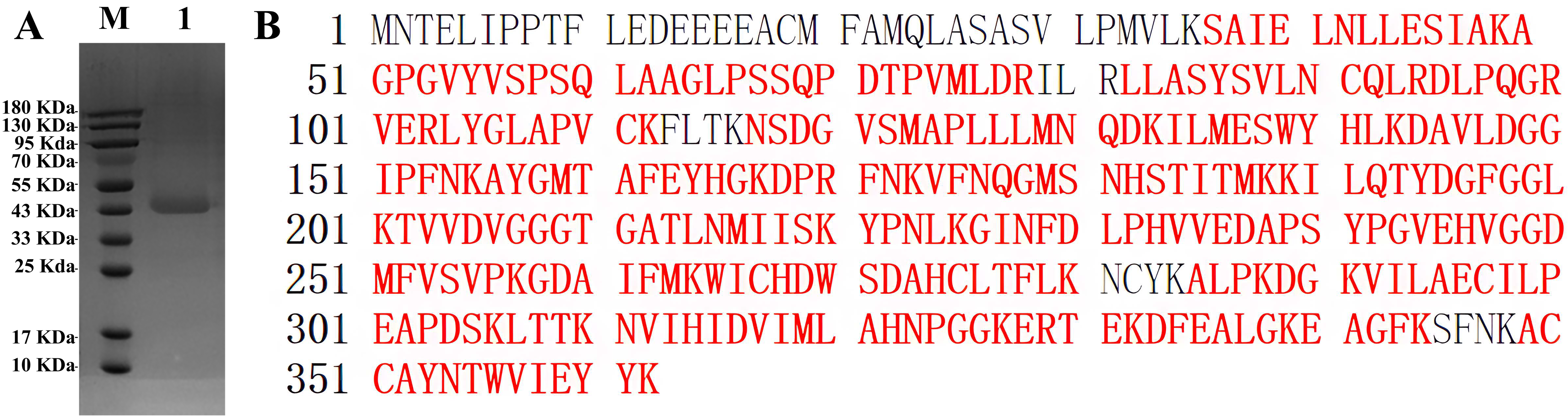


Supplementary Figure 1. Analysis of protein profiling results of *LcCOMT*. (A) Induced expression results of*LcCOMT* protein and (B) MS/MS identification of LcCOMT protein.


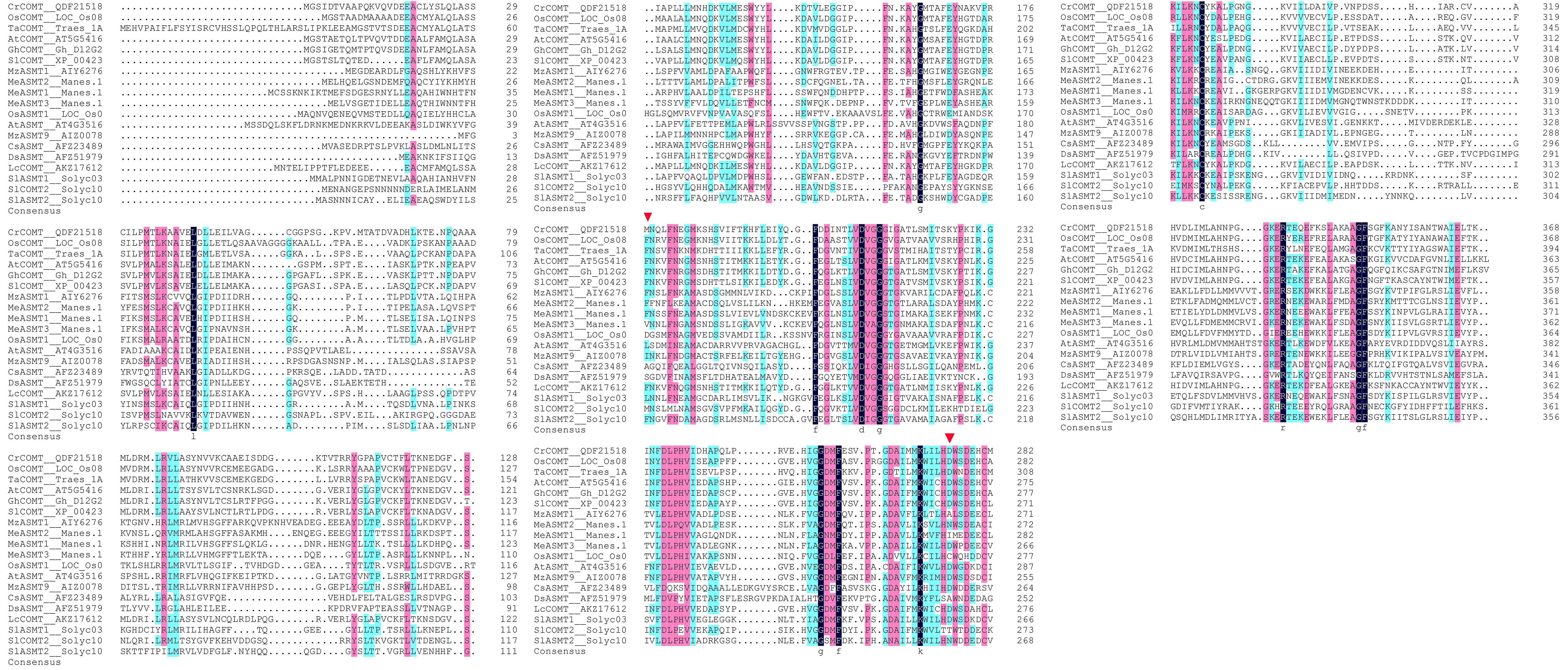


Supplementary Figure 2. Amino acid alignment among COMT/ASMTs.

Supplementary Table 1. The homology of LcCOMT to ASMT/COMT proteins from other plant species.

| Members | Accession number | Identity with LcCOMT (amino acid) |
| --- | --- | --- |
| OsASMT | LOC_Os09g17560 | 27% |
| AtASMT | AT4G35160 | 33% |
| MzASMT1 | AIY62760 | 31% |
| MeASMT1 | Manes.13G140900 | 30% |
| MeASMT2 | Manes.17G050500 | 30% |
| MeASMT3 | Manes.13G140500 | 31% |
| SlASMT1 | Solyc03g097700 | 34% |
| SlASMT2 | Solyc10g074910.1.1 | 32% |
| MzASMT9 | AIZ00789 | 41% |
| DsASMT | AFZ51979 | 31% |
| CsASMT | AFZ23489 | 33% |
| AtCOMT | AT5G54160 | 73% |
| CrCOMT | QDF21518 | 58% |
| GhCOMT | Gh_D12G2714 | 76% |
| OsCOMT | LOC_Os08g06100 | 59% |
| SlCOMT1 | XP_004235028 | 77% |
| SlCOMT2 | Solyc10g085830.1 | 39% |
| TaCOMT | Traes_1AL_D9035D5E0 | 54% |

Abbreviation: Os, *Oryza satica*; At, *Arabidopsis thaliana*; Mz, *Malus zumi*; Me, *Manihot esculenta*; Sl, *Solanum lycopersicum*; Cr, *Carexrigescens*; Ta, *Triticum aestivum*; Gh, *Gossypium hirsutum*; Cs, *Cylindrospermumstagnale* PCC 7417; Ds, *Dactylococcopsis salina* PCC 8305.

Supplementary Table 2. Relative activities of LcCOMT, LcCOMT-F171A and LcCOMT-D269A proteins for various substrates, respectively.

| Enzymes | Substrates | |
| --- | --- | --- |
|  | caffeic acid | N-acetylserotonin |
| LcCOMT | 100% | 100% |
| LCCOMT-F171A | 18.10% | 89.14% |
| LCCOMT-D269A | 5.71% | 83.44% |

Supplementary Table 3. Primers used in the paper.

| Primer | Sequence (5’-3’) |
| --- | --- |
| LcCOMT-KpnI-F | GCG GGT CGA C*GG TAC C***AT G**AA TAC GGA GCT GAT C |
| LcCOMT-KpnI-R | TAG ACA TAT G*GG TAC C***TT A**TT TAT AAT ATT CAA TAA CCC AAG TG |
| LcCOMTqFP | ATT GGA GCG ATG CAC ATT |
| LcCOMTqRP | TTT TCC GCC AGG ATT ATC |
| 18SFP | GCG ACG TTC GCT CTC TAT CCA TAC |
| 18SRP | TGG TTC ACG GGA TTC TGC AA |
| 35S-F | GAC GCA CAA TCC CAC TAT CC |
| LcCOMT-R | TTA TTT ATA ATA TTC AAT AAC CCA AGT G |
| LcCOMT-F171A-F | CCC AGA GCT AAC AAA GTC TTT AAC CAG GGA ATG TCC |
| LcCOMT-F171A-R | TGT TAG CTC TGG GAT CTT TGC CAT GGT ACT CA |
| LcCOMT-D269A-F | TGT CAC GCT TGG AGC GAT GCA CAT TGT CTG ACA TT |
| LcCOMT-D269A-R | GCT CCA AGC GTG ACA TAT CCA CTT CAT GAA AAT AGC ATC |
